# Crystal structure of 3-({[(thio­phen-2-yl)methyl­idene]hydrazin­yl}carbon­yl)pyridinium chloride dihydrate

**DOI:** 10.1107/S1600536814017565

**Published:** 2014-08-06

**Authors:** Thangayyah Chandrasekaran, Mani Suresh, John Josephine Novina, Mohamed Khan Syed Ali Padusha, Gopalsamy Vasuki, Balasubramani Kasthuri

**Affiliations:** a PG & Research Department of Chemistry, Jamal Mohamed College (Autonomous), Tiruchirappalli-20, India; b Department of Physics, Idhaya College for Women, Kumbakonam-1, India; c Department of Physics, Kunthavai Naachiar Govt. Arts College (W) (Autonomous), Thanjavur-7, India; d Department of Chemistry, Govt. Arts College (Autonomous), Thanthonimalai, Karur-5, India

**Keywords:** crystal structure, pyridinium chloride salt, hydrogen bonding, hydrazone derivatives

## Abstract

In the title compound, C_11_H_10_N_3_OS^+^·Cl^−^·2H_2_O, the organic cation exhibits a dihedral angle of 21.26 (8)° between the mean planes of the pyridine and thio­phene rings, and dihedral angles of 15.11 (9) and 6.49 (9)° between the mean planes of the hydrazide moiety and the pyridine and thio­phene rings, respectively. In the crystal, the organic cation, the chloride counter-anion and the two water mol­ecules of crystallization are linked through an intricate hydrogen-bonding network consisting of O—H⋯O, O—H⋯N, N—H⋯Cl, C—H⋯Cl, C—H⋯O, N—H⋯O, O—H⋯Cl and C—H⋯S inter­actions that consolidate a three-dimensional network.

## Related literature   

For structures of related hydrazone derivatives, see: Cheng *et al.* (2008 ▶); Jing *et al.* (2007 ▶); Novina *et al.* (2013 ▶, 2014 ▶). For the biological activity of hydrazones, see: Babahan *et al.* (2013 ▶); Kaplancikli *et al.* (2012 ▶). For graph-set notation, see: Bernstein *et al.* (1995 ▶).
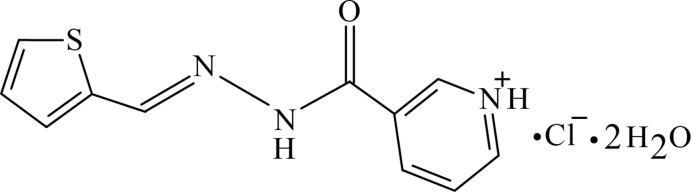


## Experimental   

### Crystal data  

C_11_H_10_N_3_OS^+^·Cl^−^·2H_2_O
*M* *_r_* = 303.76
Triclinic, 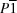

*a* = 7.8781 (7) Å
*b* = 8.6928 (7) Å
*c* = 11.0999 (10) Å
α = 67.361 (4)°
β = 78.210 (4)°
γ = 77.119 (4)°
*V* = 677.97 (10) Å^3^
*Z* = 2
Mo *K*α radiation
μ = 0.44 mm^−1^
*T* = 296 K
0.35 × 0.30 × 0.30 mm

### Data collection  

Bruker APEXII CCD diffractometer
Absorption correction: multi-scan (*SADABS*; Bruker, 2004 ▶) *T*_min_ = 0.860, *T*_max_ = 0.879
5444 measured reflections
3222 independent reflections
2761 reflections with *I* > 2σ(*I*)
*R*_int_ = 0.016

### Refinement  

*R*[*F* ^2^ > 2σ(*F* ^2^)] = 0.037
*wR*(*F* ^2^) = 0.119
*S* = 1.05
3222 reflections
192 parameters
6 restraints
H atoms treated by a mixture of independent and constrained refinement
Δρ_max_ = 0.32 e Å^−3^
Δρ_min_ = −0.40 e Å^−3^

### 

Data collection: *APEX2* (Bruker, 2004 ▶); cell refinement: *SAINT* (Bruker, 2004 ▶); data reduction: *SAINT*; program(s) used to solve structure: *SIR92* (Altomare *et al.*, 1993 ▶); program(s) used to refine structure: *SHELXL97* (Sheldrick, 2008 ▶); molecular graphics: *ORTEP-3 for Windows* (Farrugia, 2012 ▶) and *Mercury* (Macrae *et al.*, 2008 ▶); software used to prepare material for publication: *PLATON* (Spek, 2009 ▶) and *publCIF* (Westrip, 2010 ▶).

## Supplementary Material

Crystal structure: contains datablock(s) I, global. DOI: 10.1107/S1600536814017565/wm5042sup1.cif

Structure factors: contains datablock(s) I. DOI: 10.1107/S1600536814017565/wm5042Isup2.hkl

Supporting information file. DOI: 10.1107/S1600536814017565/wm5042Isup3.cml

**Fig. 1.**
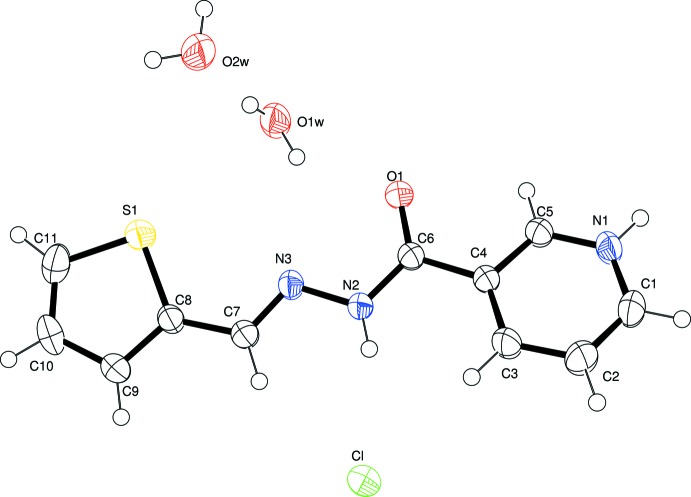
The molecular structure of the title compound, with the atom labelling. Displacement ellipsoids are drawn at the 50% probability level.

. DOI: 10.1107/S1600536814017565/wm5042fig1.tif
The mol­ecular structure of the title compound, with the atom labelling. Displacement ellipsoids are drawn at the 50% probability level.

**Fig. 2.**
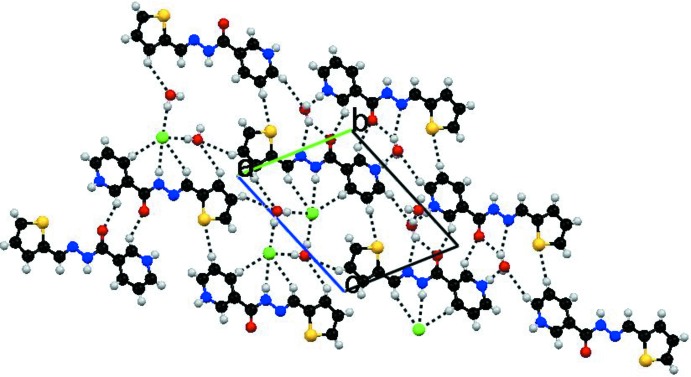
The crystal packing of the title compound viewed along the a axis. Hydrogen bonds are shown as dashed lines. R44(10), R22(10), R21(6), R21(7), R32(8) R33(7) and R33(10) ring motifs (Bernstein et al., 1995) are observed in the packing.

Click here for additional data file.a et al. . DOI: 10.1107/S1600536814017565/wm5042fig2.tif
The crystal packing of the title compound viewed along the *a* axis. Hydrogen bonds are shown as dashed lines. 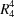
(10), 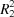
(10), 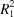
(6), 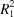
(7), 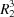
(8) 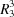
(7) and 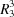
(10) ring motifs (Bernstein *et al.*, 1995) are observed in the packing.

CCDC reference: 1017163

Additional supporting information:  crystallographic information; 3D view; checkCIF report

## Figures and Tables

**Table 1:** Hydrogen-bond geometry (Å, °)

*D*—H⋯*A*	*D*—H	H⋯*A*	*D*⋯*A*	*D*—H⋯*A*
N1—H1*N*1⋯O1*W* ^i^	0.86	1.80	2.659 (2)	176
N2—H2*N*2⋯Cl	0.84 (2)	2.59 (2)	3.4011 (14)	163 (2)
O1*W*—H1*O*1⋯O1	0.87 (2)	2.11 (2)	2.8465 (18)	142 (2)
O1*W*—H1*O*1⋯N3	0.87 (2)	2.50 (2)	3.2648 (19)	148 (2)
O2*W*—H2*O*2⋯Cl^ii^	0.83 (3)	2.41 (3)	3.2305 (18)	171 (3)
O2*W*—H1*O*2⋯Cl^iii^	0.85 (2)	2.37 (2)	3.2102 (16)	171 (2)
O1*W*—H2*O*1⋯O2*W*	0.86 (2)	1.91 (2)	2.764 (2)	170 (3)
C2—H2⋯S1^iv^	0.93	2.71	3.6359 (19)	179
C3—H3⋯Cl	0.93	2.72	3.629 (2)	166
C5—H5⋯O1^i^	0.93	2.41	3.207 (2)	143

Symmetry codes: (i) 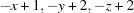; (ii) 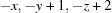; (iii) 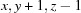; (iv) 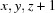.

## supplementary crystallographic information

### S1. Experimental

Thiophene-2-carboxaldehyde (1.2 ml, 0.01 mol) was added to an ethanolic solution of nicotinicacid hydrazide (1.37 g, 0.01 mol). After the addition was complete, the reaction mixture was stirred thoroughly at 273 K. To this mixture concentrated hydrochloric acid (five drops) was added and stirred. The reaction mixture was kept at this temperature for 30 min. On completion of the reaction, the resulting solid mass was seperated, filtered, dried and washed with diethylether. A pale yellow solid was obtained that was recrystallized from ethanol [yield: 82%].

### S2. Refinement

The H atoms of the solvent water molecules and of the hydrazide moiety were located in a difference map and were refined freely. All other H atoms were placed in geometrically idealized positions and constrained to ride on their parent atoms with C—H = 0.93 Å, N—H = 0.86 Å and with *U*_iso_(H) = 1.2*U*_eq_(C, N).

### Crystal data

**Table:**

C_11_H_10_N_3_OS^+^·Cl^−^·2H_2_O	*Z* = 2
*M**_r_* = 303.76	*F*(000) = 316
Triclinic, *P*1	*D*_x_ = 1.488 Mg m^−^^3^
Hall symbol: -P 1	Mo *K*α radiation, λ = 0.71073 Å
*a* = 7.8781 (7) Å	Cell parameters from 3509 reflections
*b* = 8.6928 (7) Å	θ = 2.6–28.0°
*c* = 11.0999 (10) Å	µ = 0.44 mm^−^^1^
α = 67.361 (4)°	*T* = 296 K
β = 78.210 (4)°	Block, pale yellow
γ = 77.119 (4)°	0.35 × 0.30 × 0.30 mm
*V* = 677.97 (10) Å^3^	

### Data collection

**Table:**

Bruker APEXII CCD diffractometer	3222 independent reflections
Radiation source: fine-focus sealed tube	2761 reflections with *I* > 2σ(*I*)
Graphite monochromator	*R*_int_ = 0.016
ω and φ scan	θ_max_ = 28.2°, θ_min_ = 2.6°
Absorption correction: multi-scan (*SADABS*; Bruker, 2004)	*h* = −9→10
*T*_min_ = 0.860, *T*_max_ = 0.879	*k* = −7→11
5444 measured reflections	*l* = −14→14

### Refinement

**Table:**

Refinement on *F*^2^	Primary atom site location: structure-invariant direct methods
Least-squares matrix: full	Secondary atom site location: difference Fourier map
*R*[*F*^2^ > 2σ(*F*^2^)] = 0.037	Hydrogen site location: inferred from neighbouring sites
*wR*(*F*^2^) = 0.119	H atoms treated by a mixture of independent and constrained refinement
*S* = 1.05	*w* = 1/[σ^2^(*F*_o_^2^) + (0.076*P*)^2^ + 0.1235*P*] where *P* = (*F*_o_^2^ + 2*F*_c_^2^)/3
3222 reflections	(Δ/σ)_max_ = 0.001
192 parameters	Δρ_max_ = 0.32 e Å^−^^3^
6 restraints	Δρ_min_ = −0.40 e Å^−^^3^

### Special details

**Table:**

Geometry. All e.s.d.'s (except the e.s.d. in the dihedral angle between two l.s. planes) are estimated using the full covariance matrix. The cell e.s.d.'s are taken into account individually in the estimation of e.s.d.'s in distances, angles and torsion angles; correlations between e.s.d.'s in cell parameters are only used when they are defined by crystal symmetry. An approximate (isotropic) treatment of cell e.s.d.'s is used for estimating e.s.d.'s involving l.s. planes.
Refinement. Refinement of *F*^2^ against ALL reflections. The weighted *R*-factor *wR* and goodness of fit *S* are based on *F*^2^, conventional *R*-factors *R* are based on *F*, with *F* set to zero for negative *F*^2^. The threshold expression of *F*^2^ > σ(*F*^2^) is used only for calculating *R*-factors(gt) *etc*. and is not relevant to the choice of reflections for refinement. *R*-factors based on *F*^2^ are statistically about twice as large as those based on *F*, and *R*- factors based on ALL data will be even larger.

### Fractional atomic coordinates and isotropic or equivalent isotropic displacement parameters (Å^2^)

**Table:**

	*x*	*y*	*z*	*U*_iso_*/*U*_eq_	
S1	0.17836 (6)	0.47295 (5)	0.80609 (4)	0.04223 (14)	
Cl	0.17151 (7)	0.25503 (5)	1.42848 (4)	0.05065 (16)	
O1	0.38027 (16)	0.80909 (14)	1.00852 (10)	0.0399 (3)	
O1W	0.3488 (2)	0.82526 (19)	0.75281 (13)	0.0514 (3)	
N2	0.31200 (17)	0.55367 (15)	1.14192 (12)	0.0315 (3)	
N3	0.27791 (16)	0.52406 (15)	1.03627 (12)	0.0306 (3)	
O2W	0.0187 (2)	0.9549 (2)	0.67510 (14)	0.0640 (4)	
N1	0.47813 (17)	0.91856 (17)	1.31029 (13)	0.0367 (3)	
H1N1	0.5298	1.0037	1.2922	0.044*	
C9	0.1401 (2)	0.18261 (19)	0.98202 (16)	0.0367 (3)	
H9	0.1335	0.0925	1.0618	0.044*	
C4	0.37426 (18)	0.74005 (17)	1.23659 (14)	0.0287 (3)	
C6	0.35574 (18)	0.70370 (17)	1.11885 (13)	0.0284 (3)	
C5	0.4561 (2)	0.87569 (19)	1.21193 (15)	0.0329 (3)	
H5	0.4964	0.9381	1.1257	0.040*	
C7	0.23292 (19)	0.38064 (18)	1.06447 (14)	0.0318 (3)	
H7	0.2300	0.3041	1.1506	0.038*	
C8	0.18671 (19)	0.33584 (18)	0.96480 (14)	0.0306 (3)	
C11	0.1187 (2)	0.3284 (3)	0.76111 (18)	0.0471 (4)	
H11	0.0986	0.3481	0.6763	0.057*	
C10	0.1041 (2)	0.1815 (2)	0.86327 (19)	0.0466 (4)	
H10	0.0732	0.0886	0.8558	0.056*	
C3	0.3133 (3)	0.6516 (2)	1.36593 (16)	0.0471 (4)	
H3	0.2565	0.5600	1.3860	0.056*	
C2	0.3378 (3)	0.7008 (3)	1.46513 (17)	0.0610 (6)	
H2	0.2962	0.6429	1.5522	0.073*	
C1	0.4229 (3)	0.8340 (2)	1.43523 (17)	0.0481 (4)	
H1	0.4421	0.8655	1.5019	0.058*	
H2N2	0.298 (3)	0.482 (3)	1.218 (2)	0.057 (6)*	
H1O1	0.337 (4)	0.778 (3)	0.8380 (17)	0.086 (9)*	
H2O2	−0.019 (4)	0.898 (3)	0.644 (3)	0.093 (10)*	
H1O2	0.047 (3)	1.041 (3)	0.610 (2)	0.078 (8)*	
H2O1	0.243 (2)	0.854 (3)	0.734 (3)	0.079 (9)*	

### Atomic displacement parameters (Å^2^)

**Table:**

	*U*^11^	*U*^22^	*U*^33^	*U*^12^	*U*^13^	*U*^23^
S1	0.0576 (3)	0.0397 (2)	0.0315 (2)	−0.01844 (18)	−0.00473 (17)	−0.00956 (16)
Cl	0.0716 (3)	0.0443 (3)	0.0392 (2)	−0.0295 (2)	−0.0095 (2)	−0.00611 (18)
O1	0.0609 (7)	0.0349 (5)	0.0264 (5)	−0.0208 (5)	−0.0068 (5)	−0.0060 (4)
O1W	0.0708 (9)	0.0549 (8)	0.0352 (7)	−0.0351 (7)	−0.0011 (6)	−0.0127 (6)
N2	0.0442 (7)	0.0286 (6)	0.0247 (6)	−0.0124 (5)	−0.0048 (5)	−0.0091 (5)
N3	0.0361 (6)	0.0319 (6)	0.0282 (6)	−0.0097 (5)	−0.0034 (5)	−0.0135 (5)
O2W	0.0985 (12)	0.0610 (9)	0.0386 (7)	−0.0414 (8)	−0.0182 (7)	−0.0039 (6)
N1	0.0451 (7)	0.0370 (7)	0.0360 (7)	−0.0189 (6)	−0.0028 (5)	−0.0161 (5)
C9	0.0448 (8)	0.0295 (7)	0.0375 (8)	−0.0108 (6)	−0.0059 (6)	−0.0108 (6)
C4	0.0331 (7)	0.0275 (6)	0.0272 (7)	−0.0086 (5)	−0.0032 (5)	−0.0099 (5)
C6	0.0307 (7)	0.0284 (6)	0.0276 (7)	−0.0080 (5)	−0.0033 (5)	−0.0099 (5)
C5	0.0384 (7)	0.0332 (7)	0.0294 (7)	−0.0140 (6)	−0.0017 (5)	−0.0104 (6)
C7	0.0377 (7)	0.0298 (7)	0.0293 (7)	−0.0087 (5)	−0.0032 (5)	−0.0107 (5)
C8	0.0342 (7)	0.0297 (7)	0.0306 (7)	−0.0096 (5)	−0.0021 (5)	−0.0125 (5)
C11	0.0487 (9)	0.0635 (11)	0.0410 (9)	−0.0144 (8)	−0.0078 (7)	−0.0276 (8)
C10	0.0490 (9)	0.0458 (9)	0.0606 (11)	−0.0173 (7)	−0.0065 (8)	−0.0307 (8)
C3	0.0756 (13)	0.0421 (9)	0.0295 (8)	−0.0334 (9)	0.0031 (7)	−0.0111 (7)
C2	0.1039 (17)	0.0616 (12)	0.0248 (8)	−0.0459 (12)	0.0060 (9)	−0.0126 (8)
C1	0.0703 (12)	0.0516 (10)	0.0336 (8)	−0.0218 (9)	−0.0054 (8)	−0.0214 (7)

### Geometric parameters (Å, º)

**Table:**

S1—C11	1.6999 (18)	C9—C10	1.408 (2)
S1—C8	1.7101 (15)	C9—H9	0.9300
Cl—Cl	0.0000 (12)	C4—C5	1.3780 (19)
O1—C6	1.2226 (17)	C4—C3	1.384 (2)
O1W—H1O1	0.868 (17)	C4—C6	1.4985 (19)
O1W—H2O1	0.857 (16)	C5—H5	0.9300
N2—C6	1.3389 (18)	C7—C8	1.438 (2)
N2—N3	1.3804 (17)	C7—H7	0.9300
N2—H2N2	0.83 (2)	C11—C10	1.352 (3)
N3—C7	1.2764 (18)	C11—H11	0.9300
O2W—H2O2	0.837 (17)	C10—H10	0.9300
O2W—H1O2	0.851 (16)	C3—C2	1.386 (2)
N1—C1	1.328 (2)	C3—H3	0.9300
N1—C5	1.3344 (19)	C2—C1	1.364 (3)
N1—H1N1	0.8600	C2—H2	0.9300
C9—C8	1.393 (2)	C1—H1	0.9300
			
C11—S1—C8	92.07 (8)	N3—C7—C8	120.85 (13)
H1O1—O1W—H2O1	104 (2)	N3—C7—H7	119.6
C6—N2—N3	117.65 (12)	C8—C7—H7	119.6
C6—N2—H2N2	122.4 (16)	C9—C8—C7	126.41 (14)
N3—N2—H2N2	119.8 (16)	C9—C8—S1	111.11 (11)
C7—N3—N2	114.96 (12)	C7—C8—S1	122.47 (11)
H2O2—O2W—H1O2	106 (2)	C10—C11—S1	111.95 (13)
C1—N1—C5	122.12 (13)	C10—C11—H11	124.0
C1—N1—H1N1	118.9	S1—C11—H11	124.0
C5—N1—H1N1	118.9	C11—C10—C9	113.50 (15)
C8—C9—C10	111.34 (14)	C11—C10—H10	123.2
C8—C9—H9	124.3	C9—C10—H10	123.2
C10—C9—H9	124.3	C4—C3—C2	119.31 (15)
C5—C4—C3	118.07 (14)	C4—C3—H3	120.3
C5—C4—C6	116.35 (12)	C2—C3—H3	120.3
C3—C4—C6	125.57 (13)	C1—C2—C3	120.10 (16)
O1—C6—N2	123.36 (13)	C1—C2—H2	120.0
O1—C6—C4	119.87 (12)	C3—C2—H2	120.0
N2—C6—C4	116.77 (12)	N1—C1—C2	119.56 (15)
N1—C5—C4	120.82 (13)	N1—C1—H1	120.2
N1—C5—H5	119.6	C2—C1—H1	120.2
C4—C5—H5	119.6		

### Hydrogen-bond geometry (Å, º)

**Table:**

*D*—H···*A*	*D*—H	H···*A*	*D*···*A*	*D*—H···*A*
N1—H1*N*1···O1*W*^i^	0.86	1.80	2.659 (2)	176
N2—H2*N*2···Cl	0.84 (2)	2.59 (2)	3.4011 (14)	163 (2)
O1*W*—H1*O*1···O1	0.87 (2)	2.11 (2)	2.8465 (18)	142 (2)
O1*W*—H1*O*1···N3	0.87 (2)	2.50 (2)	3.2648 (19)	148 (2)
O2*W*—H2*O*2···Cl^ii^	0.83 (3)	2.41 (3)	3.2305 (18)	171 (3)
O2*W*—H1*O*2···Cl^iii^	0.85 (2)	2.37 (2)	3.2102 (16)	171 (2)
O1*W*—H2*O*1···O2*W*	0.86 (2)	1.91 (2)	2.764 (2)	170 (3)
C2—H2···S1^iv^	0.93	2.71	3.6359 (19)	179
C3—H3···Cl	0.93	2.72	3.629 (2)	166
C5—H5···O1^i^	0.93	2.41	3.207 (2)	143

Symmetry codes: (i) −*x*+1, −*y*+2, −*z*+2; (ii) −*x*, −*y*+1, −*z*+2; (iii) *x*, *y*+1, *z*−1; (iv) *x*, *y*, *z*+1.

## References

[bb1] Altomare, A., Cascarano, G., Giacovazzo, C. & Guagliardi, A. (1993). *J. Appl. Cryst.* **26**, 343–350.

[bb2] Babahan, I., Coban, E. P. & Biyik, H. (2013). *Maejo Int. J. Sci. Technol.* **7**, 26–41.

[bb3] Bernstein, J., Davis, R. E., Shimoni, L. & Chang, N.-L. (1995). *Angew. Chem. Int. Ed. Engl.* **34**, 1555–1573.

[bb4] Bruker (2004). *APEX2*, *SAINT* and *SADABS*, Bruker AXS Inc., Madison, Wisconsin, USA.

[bb5] Cheng, H., Djukic, B., Harrington, L. E., Britten, J. F. & Lemaire, M. T. (2008). *Acta Cryst.* E**64**, o719. 10.1107/S1600536808004960 PMC2961063 21202109

[bb6] Farrugia, L. J. (2012). *J. Appl. Cryst.* **45**, 849–854.

[bb7] Jing, Z.-L., Yu, M. & Chen, X. (2007). *Acta Cryst.* E**63**, o4029.

[bb8] Kaplancikli, Z. A., Altintop, M. D., Özdemir, A., Turan-Zitouni, G., Khan, S. I. & Tabanca, N. (2012). *Lett. Drug Des. Discov* **9**, 310–315.

[bb9] Macrae, C. F., Bruno, I. J., Chisholm, J. A., Edgington, P. R., McCabe, P., Pidcock, E., Rodriguez-Monge, L., Taylor, R., van de Streek, J. & Wood, P. A. (2008). *J. Appl. Cryst.* **41**, 466–470.

[bb10] Novina, J. J., Vasuki, G., Suresh, M. & Padusha, M. S. A. (2013). *Acta Cryst.* E**69**, o1177–o1178. 10.1107/S1600536813017406 PMC3770434 24046719

[bb11] Novina, J. J., Vasuki, G., Suresh, M. & Padusha, M. S. A. (2014). *Acta Cryst.* E**70**, o793–o794. 10.1107/S1600536814013798 PMC4120580 25161576

[bb12] Sheldrick, G. M. (2008). *Acta Cryst.* A**64**, 112–122. 10.1107/S0108767307043930 18156677

[bb13] Spek, A. L. (2009). *Acta Cryst.* D**65**, 148–155. 10.1107/S090744490804362X PMC2631630 19171970

[bb14] Westrip, S. P. (2010). *J. Appl. Cryst.* **43**, 920–925.

