# Supplementary figures and images for: Crystal structure of 3-({[(thio­phen-2-yl)methyl­idene]hydrazin­yl}carbon­yl)pyridinium chloride dihydrate
Source: Acta Crystallogr Sect E Struct Rep Online. 2014 Aug 6;70(Pt 9):o976–7. doi: 10.1107/S1600536814017565 (PMC4186149; doi:10.1107/S1600536814017565)

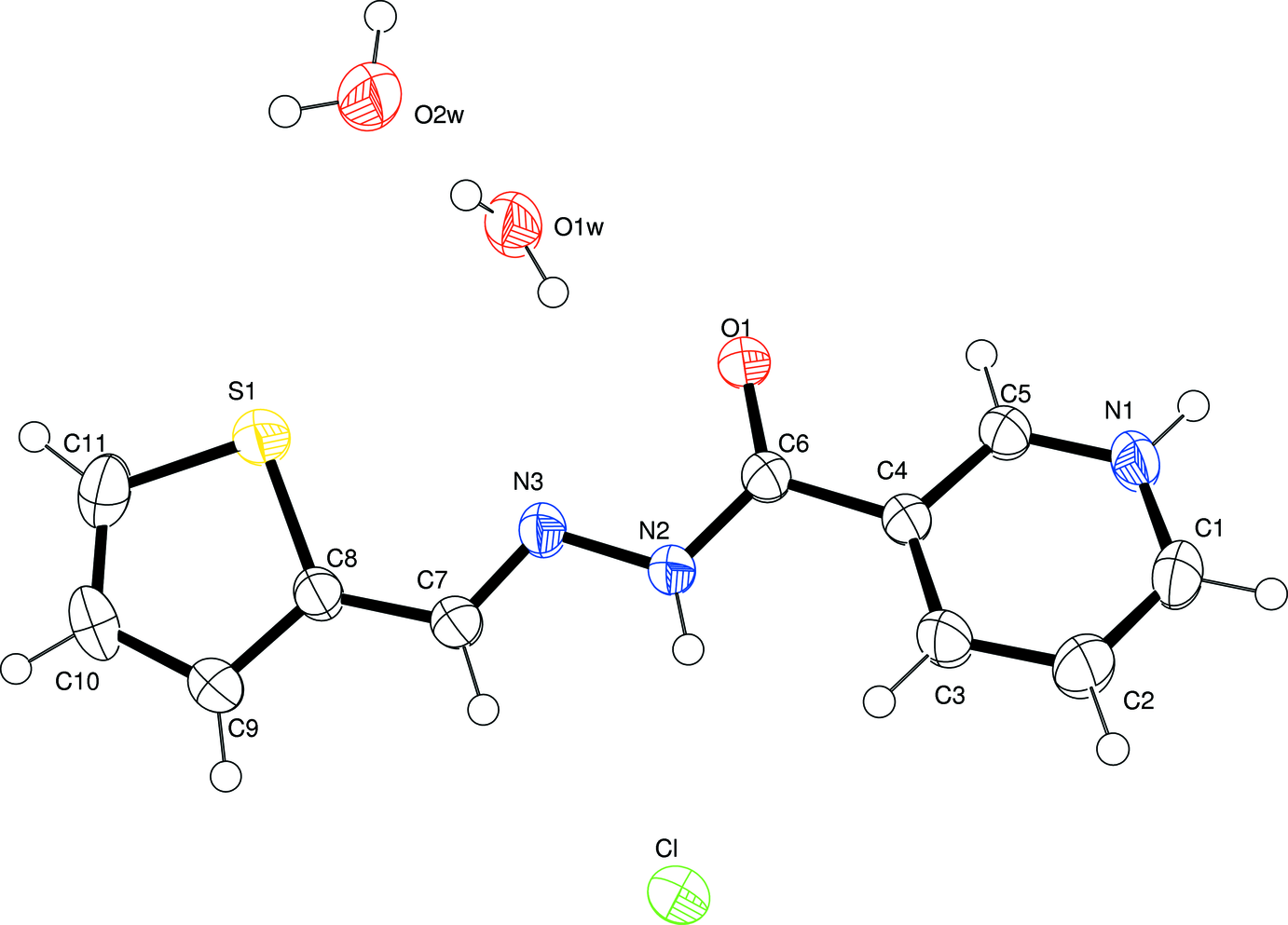

Supplement: Supplementary file 4 [file e-70-0o976-fig1.tif]

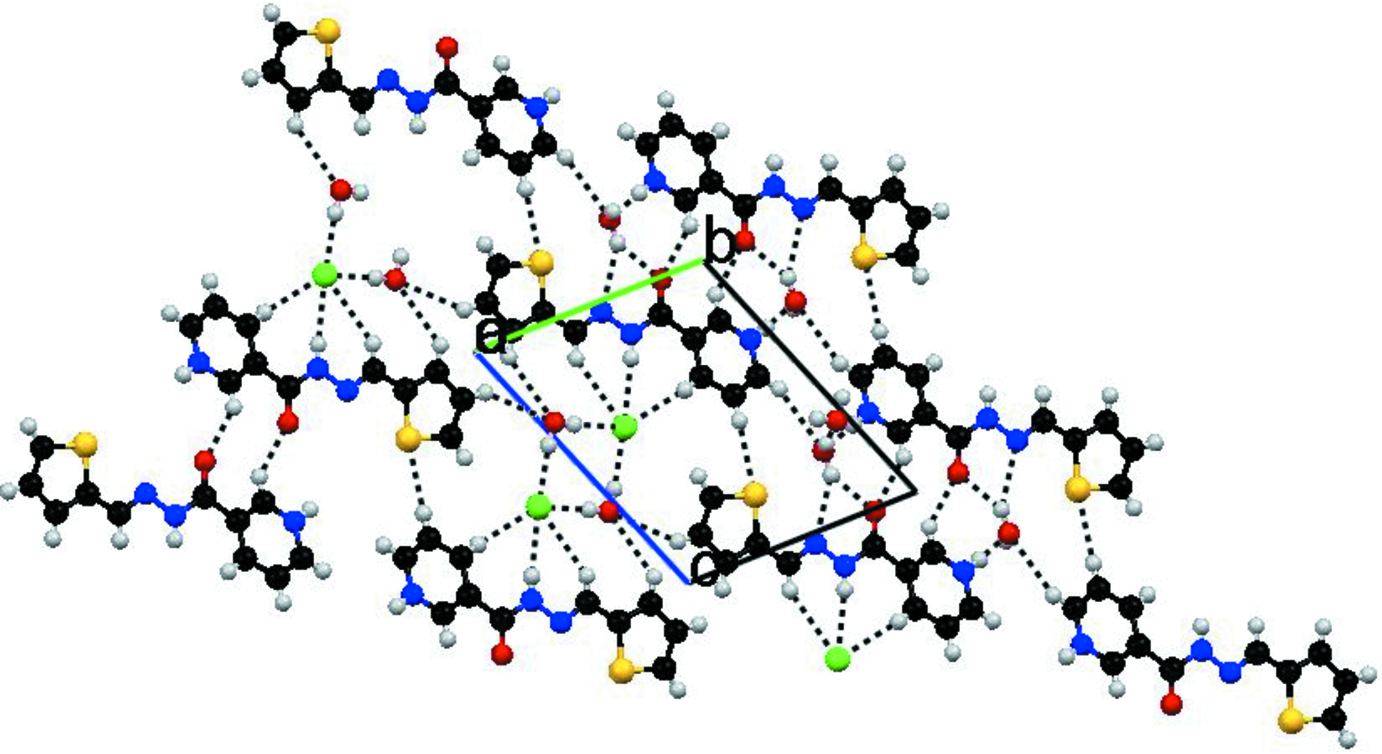

Supplement: Supplementary file 5 [file e-70-0o976-fig2.tif]
